# Current status and perspectives of hematopoietic cell transplantation in patients with paroxysmal nocturnal hemoglobinuria

**DOI:** 10.3389/fimmu.2024.1521484

**Published:** 2025-01-07

**Authors:** Marek Ussowicz, Dawid Przystupski, Patrycja Mensah-Glanowska, Agnieszka Piekarska

**Affiliations:** ^1^ Department of Paediatric Bone Marrow Transplantation, Oncology and Hematology, Wroclaw Medical University; Supraregional Centre of Paediatric Oncology “Cape of Hope”, Wrocław, Poland; ^2^ Department of Hematology, Jagiellonian University Collegium Medicum, University Hospital in Cracow, Kraków, Poland; ^3^ Department of Hematology and Transplantology, Medical University of Gdansk, Gdansk, Poland

**Keywords:** PNH, aplastic anemia, hematopoietic cell transplantation, paroxysmal nocturnal hemoglobinuria, conditioning

## Abstract

**Background:**

Paroxysmal nocturnal hemoglobinuria (PNH) is a rare complement-driven acquired hemolytic anemia with specific presentations of hemoglobinuria, abdominal pain, fatigue, and thrombosis.

**Objective:**

To review the current therapeutic strategies for PNH, including anti-complement therapy and allogeneic hematopoietic cell transplantation (alloHCT), focusing on the tailoring of the approach to the disease subtype.

**Results:**

The outcome of alloHCT varies depending on disease severity, thrombotic history, and response to prior therapies. Non-transplant PNH therapies include anti-C5 monoclonal antibodies that reduce terminal complement activation (eculizumab, ravulizumab, and crovalimab) and proximal complement pathway inhibitors such as pegcetacoplan (C3 inhibitor), iptacopan (complement factor B inhibitor), and danicopan (complement factor D inhibitor). Although complement inhibitors have revolutionized treatment, alloHCT remains the only curative therapy, particularly for patients who are refractory to medical management or have severe cytopenia. This review outlines the conditioning regimens used in alloHCT and summarizes recent studies showing that overall survival rates improve with less toxic conditioning protocols.

**Conclusions:**

AlloHCT can be used to manage PNH, particularly in patients who are resistant to or without access to complement-targeted therapies. Any potential cure offered by alloHCT must be counterbalanced by the significant procedure risks, including graft-versus-host disease and transplant-related mortality, particularly in patients with comorbidities. In the case of severe aplastic anemia with an associated PNH clone, immunoablative protocols based on anti-thymocyte globulin serotherapy with fludarabine and cyclophosphamide are recommended. The use of reduced toxicity protocols with fludarabine has been well-documented in patients with classic PNH. A treosulfan/fludarabine-based regimen is recommended; however, there is no consensus on optimal drug selection.

## Introduction

### Pathophysiology and diagnostics of PNH

Glycosylphosphatidylinositol (GPI) anchoring of proteins is a conserved posttranslational modification in eukaryotes that enables the sorting of GPI-anchored proteins in the secretory and endocytic pathways (1). Constitutional phosphatidylinositol glycan biosynthesis class A (PIGA) protein mutations are rare and have been implicated in severe neurological development impairments, cardiac anomalies, and a high frequency of spontaneous death, especially in childhood (2, 3).

Somatic PIGA mutations, and less commonly, phosphatidylinositol glycan anchor biosynthesis class B (PIGB) and T (PIGT) mutations, are responsible for paroxysmal nocturnal hemoglobinuria (PNH), a complement-driven acquired hemolytic anemia that often manifests as hemoglobinuria, abdominal pain, smooth muscle dystonia, fatigue, and thrombosis (4–6). It has been hypothesized that an autoimmune attack in aplastic anemia (AA) on normal stem cells targets a GPI-linked molecule and therefore preferentially spares PNH stem cells, or that PNH cells are resistant to proapoptotic signals and thus have a growth or survival advantage (7, 8). The risk of clonal malignancy (myelodysplastic syndrome [MDS] and acute myeloid leukemia [AML]) in PNH is relatively low at an estimated 3% to 10% (9–11). Patients with PNH that evolves into MDS/AML usually present with monosomy 7, and mutations in the ASXL1, SETBP1, RUNX1, or RAS pathways (12).

There are two clinically relevant PNH subtypes that modify the therapeutic approach at the time of diagnosis: classic PNH (cPNH), which manifests with hemolytic anemia and thrombosis, and a PNH clone associated with MDS or AA (AA/PNH), with a predominant symptomatology of underlying bone marrow failure (BMF), often without overt hemolysis (13). However, there is a notable population of patients in whom AA/PNH evolves into cPNH after different periods. Bat et al. analyzed the case histories of 319 patients diagnosed with AA at a single center between 2000 and 2017. Of these patients, 117 were diagnosed with AA without a PNH clone, 132 with AA/PNH, 26 with post-AA PNH (secondary PNH), and 44 with primary PNH. Patients were further divided into “expanders” and “stable” groups on the basis of their initial clone size and PNH clone size shifts. Initial clones containing >10% GPI-AP-deficient granulocytes were more likely to expand than those with smaller clones that remained relatively stable. Additionally, patients who received more intense immunosuppressive therapy (IST) containing anti-thymocyte globulin (ATG) were less likely to experience PNH clone expansion than those who were not treated or were treated less intensively with cyclosporin A (CsA) alone. The application of intense IST with ATG treatment has been recommended not only to improve blood counts, but also to reduce the risk of clonal expansion in patients with AA/PNH (14).

The gold standard for the modern diagnosis of PNH is the flow cytometric fluorescein-labeled aerolysin assay (FLAER), which uses proaerolysin with Alexa Fluor 488 to determine the percentage of normal (type I), partially GPI-deficient (type II), and fully GPI-deficient (type III) cells. This assay is based on the ability of aerolysin and proaerolysin to bind selectively and with a high affinity to the GPI anchor (15). Typically, the PIGA mutations responsible for type III defects are frameshift mutations, whereas those responsible for type II defects are missense mutations (16). The presence of type III cells is a hallmark of more severe symptoms resulting from intravascular hemolysis (IVH), especially in the case of a larger clone typical for cPNH. Whereas in the case of partial GPI defects characterized by type II cell occurrence, the hemolytic symptoms might be milder. In most patients, both types of defective cells have been identified at different ratios. However, in patients with AA/PNH with observed expansion of PNH clones, type III subclones are more likely to expand than type II subclones because of their probable survival advantage (14).

Historically, the initial manifestations of hemolytic symptoms and positive tests for non-immune-mediated hemolysis defined cPNH; however, with the development of modern flow cytometry and next-generation sequencing, the presence of PNH clones in different entities has been documented. Notably, PNH clones have been reported in patients with myeloproliferative neoplasms and constitutional BMF syndromes, although they are assumed to be anomalous (17–19). Evidence suggests that small PNH clones can be found in healthy individuals. However, mutations occur in differentiated progenitors rather than in hematopoietic stem cells (20). Fattizzo et al. tested 3085 adult patient samples with AA (531) or MDS (869) using high-sensitivity cytometry and identified PNH clones in 25% of the samples, including small (<10%) and very small (<1%) PNH clones (21). PNH clones, irrespective of size, were good predictors of response to IST or HCT in patients with MDS and AA. 

Although PNH clones have been reported in pediatric patients, their clinical significance has not been fully elucidated. Timeus et al. studied 85 children with acquired AA and reported a GPI-negative population in 41% of patients at diagnosis, 48% during IST, and in 45% off therapy (22). In contrast to the findings of an adult study, the therapeutic response was similar between PNH-positive and PNH-negative children. Although PNH clones are strongly associated with AA diagnosis, the management standards of AA acknowledge its presence, but do not involve it in clinical decision-making (23).

### Therapeutic background

Depending on the severity of cytopenia, age, and donor availability, patients with AA/PNH proceed to allogeneic hematopoietic cell transplantation (alloHCT) or are treated with ISTs (such as steroids, CsA, and ATG). Patients in whom pharmacotherapy has failed are referred for delayed alloHCT. Patients with cPNH and associated cytopenia and red cell transfusions can be treated with immunosuppression; however, alloHSCT has historically been the only option to prevent hemolysis-related complications. Currently available therapies for cPNH have dramatically evolved over the past decade, shifting from symptomatic management to targeted therapies aimed at complement system. This revolution began with the introduction of an anti-C5 monoclonal antibody (eculizumab) to reduce terminal complement activation. In response to the need for longer-lasting treatment, ravulizumab was developed with an extended half-life that allows for dosing every 8 weeks and is non-inferior to eculizumab, as validated in clinical studies (24, 25). Another promising C5 inhibitor, crovalimab, is designed for both subcutaneous and intravenous administration (26). Crovalimab binds to a distinct epitope on the beta subunit of C5 and has been shown to be effective in individuals of Asian descent who carry polymorphisms in C5 that affect Arg885, modifying the eculizumab and ravulizumab binding sites and leading to poor IVH control (26, 27).

One study showed that optimal clinical outcomes were not achieved for a considerable proportion of patients with PNH who received eculizumab (28). The majority (79–89%) of patients did not achieve normalized hemoglobin levels, with 76–93% having elevated bilirubin or absolute reticulocyte counts in any 24-week window, which, to a large extent, results from extravascular hemolysis (EVH). Pegcetacoplan, a pegylated peptide targeting the proximal complement protein C3, provides comprehensive control over both IVH and EVH. Clinical trials have shown that pegcetacoplan monotherapy is superior for improving hemoglobin levels and reducing transfusion dependency (29, 30). Iptacopan, an oral factor B inhibitor, targets the alternative complement pathway upstream of C3 and comprehensively inhibits complement-mediated hemolysis in monotherapy (26, 31–33). Another proximal pathway inhibitor, danicopan, is an oral factor D inhibitor designed to prevent the formation of C3 convertase and was approved as an adjuvant treatment to eculizumab or ravulizumab in PNH patients with EVH (34, 35).

Inhibition of the complement activation cascade is associated with a reduction in IVH-related complications, including thromboembolic events. However, in some patients who experience suboptimal control of IVH secondary to incomplete C5 inhibition and episodes of breakthrough intravascular hemolysis (BTH), the risk of thromboembolic complications remains. Although complement-targeted treatment does not seem to modify the mechanical evolution of the underlying bone marrow dysfunction, the data can be ambiguous. A French retrospective comparison study of 123 patients treated with eculizumab after 2005 and 191 historical controls revealed a lower cumulative incidence of AA in the eculizumab group (1% [<1 to 5]) than in the historical control cohort (10% [4 to 8]) (11). Additionally, a similar risk of clonal evolution (MDS or AML) was reported between patients receiving eculizumab (5% [2 to 11]) and historical controls diagnosed after 1985 (5% [2 to 11]). Hill et al. recently revealed that concomitant treatment with eculizumab and IST is safe and effective, regardless of the treatment sequence used (36).

Despite the remarkable advancements in medical therapy, alloHCT remains the only curative option for both cPNH and AA/PNH. This procedure is reserved for patients with cPNH who are unresponsive to complement inhibitors or those with symptomatic BMF. However, transplantation is also considered in countries with limited access to new complement-directed therapies. The potential cure offered by alloHCT must be counterbalanced with the significant risks of the procedure, including graft-versus-host disease (GVHD) and transplant-related mortality (TRM).

Each therapeutic innovation is associated with various levels of effectiveness, quality of life, disease burden, and side effect severity. This review aimed to summarize the knowledge of alloHCT in patients with a PNH clone and identify the optimal direction for future studies.

### Transplantation strategies in PNH

PNH is one of the oldest hematological entities treated with alloHCT (37). Owing to the non-malignant nature of PNH, bone marrow transplantation (BMT) is preferred over stimulated peripheral blood progenitor cell transplantation (PBPCT) to avoid immune-mediated complications. The outcomes reported in the literature span different eras, with variable patient and donor selections, BMs versus PBPCs as sources of hematopoietic cells, and diverse conditioning regimens (38). Patient characteristics and transplant outcomes are summarized in **Table 1**.

**Table 1:** Key studies on transplantation techniques and alloHCT outcomes for treating PNH.

Reference	PNH type (patient number)	HSC source	Donor type	Conditioning protocol	Thrombosis	Survival rate	Survival probability	P value
Hershko et al. (39)	cPNH (1 pt)	PBPC	twin-donor	none	none	NA	NA	NA
Szer at al (40).	AA/PNH (4 pt)	BM	MSD (3) twin-donor (1)	Cy/PCZ/ATG(1) Cy (2) None (1)	1	4-year OS	100%	NA
Endo et al. (41)	cPNH (1 pt)	BM	twin-donor	none	none	NA	NA	NA
Antin et al. (42)	AA/PNH (4 pt)	BM	MSD	Cy/PCZ/ATS (3 pt), Bu/Cy/PCZ/ATS (1 pt)	none	NA	NA	NA
Raiola et al. (43)	cPNH (7 pt)	BM	MSD	Cy (160 mg/kg) and Bu (10-14 mg/kg)	none	NA	NA	NA
Suenaga et al. (44)	cPNH (1 pt)	nonmyeloablative stem cell transplantation (NST)	MSD	cladribine 6 x 0.11 mg/kg, Bu 2 x 4 mg/kg, and rabbit ATG 2 x 2.5 mg/kg	none	NA	NA	NA
Lee et al. (45)	cPNH (5 pt)	PBPC (4 pt), BM (1 pt)	MSD (3 pt), mMUD (2 pt)	Bu 4 mg/kg/d for 2 days, Flu 30 mg/m (2)/d for 6 days, and ATG 20 mg/kg/d for 4 days (4 pt); Cy 50 mg/kg/day for 4 d and ATG 30 mg/kg/day for 3 d (1 pt)	recurrent thrombosis (3 pt)	NA	NA	NA
Chen et al. (46)	cPNH (4 pt), AA/PNH (14 pt)	PBPC +BM (14 pt), BM (2 pt), PBPC (2 pt)	MSD (7 pt), MUD (2 pt), haploidentical donor (9 pt)	Bu (9.6 mg/kg)/Cy (3.6 g/m (2)) - based regimens (13 pt); non-myeloablative regimens: Bu 6.4 mg/kg or Cy 100 mg/kg+ fludarabine 150 mg/m2+ ATG 10 mg/kg (5 pt)	transplant-associated thrombotic microangiopathy (1 pt)	5-year DFS	80.5 ± 10.2%	NA
Schcolnik-Cabrera et al. (47)	AA-PNH (6 pt)	PBSC	MSD	reduced-intensity conditioning regimen for stem cell transplantation (RIST)	none	5-year OS	83.3%	NA
Kamranzadeh Fumani et al. (48)	AA/PNH (5 pt), cPNH (8 pt)	PBSC	MSD	Bu-Cy	the presence of thrombosis (4 pt) was associated with higher mortality rates (hazard ratio = 9.77, P = 0.06)	5-year OS	74.07%	NA
Liu et al. (49)	cPNH (16 pt), AA/PNH (30 pt)	BM (3 pt), PBPC (9 pt), BM+ PBPC (34 pt)	MSD (16 pt), MUD (4 pt), UD (1 pt), haploidentical donor (25 pt)	Bu (6.4 mg/kg), Cy (50 mg/kg), and ATG (10 mg/kg) (for haplo- and MUD); fludarabine (180 mg/m^2^), Cy (100 mg/kg), and ATG (12.5 mg/kg) regimen (MSD)	none	3-year OS	100± 0.0% in cPNH, 85.7 ± 6.6% in AA/PNH	P=0.141
						3-year GFFS	100% (± 0.0) in cPNH and 78.7% (± 7.7) AA/PNH	P*=0.067*
Xia et al. (50)	cPNH (4 pt), AA/PNH (13pt)	BM+ PBPC	MSD (7 pt), haploidentical donor (10 pt)	Bu-Cy-ATG	1 pt died due to transplant-associated thrombotic microangiopathy	3-year OS	77.8% (± 15.2)	NA
Lu et al. (51)	cPNH (32 pt)	BM+ PBPC (17 pt), PBPC (15 pt)	MUD (15 pt), haploidentical donor (17 pt)	MAC: Bu 4x 3.2 mg/kg/d on days -9 to -6, Cy 2x 1.8 g/m2/d on days -5 to -4, Flu 5x30 mg/m^2^/d on days -6 to -2 (27 pt)	3 pts died due to transplant-associated thrombotic microangiopathy (TA-TMA)	3-year OS	74.1% ± 11.4% in HID, 93.3% ± 6.4% in MUD	P=0.222
				RIC: 2x Bu 3.2 mg/kg/d on days -7 to -6, Cy 4x 500 mg/m^2^/d on days -5 to -2 or Flu 4x30 mg/m^2^/d on days 5 to -2 (5 pt)		3-year GFFS	68.8% ± 11.8% in HID, 86.7% ± 8.8% in MUD	P=0.307
Hegenbart et al. (52)	cPNH (7 pt)	PBPC	MSD (2 pt), MUD (5 pt)	Flu 30 mg/m (2)/d on days -4 to -2 and 2 Gy of TBI on day 0	history of thrombosis (4 pt), Budd-Chiari syndrome (2 pt)	NA	NA	NA
Cooper et al. (53)	cPNH (17 pt), AA/PNH (38 pt)	BM (26 pt), PBPC (25 pt), umbilical cord (3 pt), BM+ PBPC (1 pt)	MSD (20 pt), MUD (20 pt), umbilical cord (3 pt), syngenic (2 pt), other (11 pt)	MAC: Bu dosed to target a serum level of 600 to 950 ng/mL and given in daily doses for 4 to 5 days (18 pt); treosulfan 42 g/m^2^ and Flu 150 mg/kg with or without 2 Gy TBI and rabbit ATG 6 mg/kg (4 pt); Cy 120 mg/kg with ATG 60 mg/kg and high-dose TBI (12.0 to 14.0 Gy) (4 pt)	refractory thrombosis (6 pt)	5-year OS	70%	NA
				RIC: Cy 200 mg/kg (1 pt); Cy 200 mg/kg with ATG 60 to 90 mg/kg (3 pt); Flu 120 mg/m^2^, Cy 100 mg/kg, ATG 90 mg/kg, and low-dose TBI (2 Gy) (2 pt); Flu 90 to 150 mg/m^2^ with low-dose TBI (2.0 to 4.0 Gy) (21 pt)				
Shasheleva et al. (54)	AA/PNH (5 pt)	PBPC (5 pt)	MUD (5 pt)	thoraco-abdominal irradiation 4-6 Gy, Cy 100 mg/kg, Flu 150 mg/m^2^ and ATG or alemtuzumab	none	1.7-year OS	100%	NA
Füreder et al. (55)	cPNH (1 pt), AA/PNH (5 pt)	NA	MUD (5 pt), HLA-identical sibling (1 pt)	NA	NA	OS	83.3%	NA
de Latour et al. (56)	cPNH (85 pt), AA/PNH (103 pt)	BM (135 pt), PBPC (71 pt)	MUD (74 pt), HLA-identical sibling (136 pt)	Cy-based in 70% and fludarabine‐based in 30%.	thrombosis (47 pt)	5-year OS	68% (± 3) in the transplanted group; 54% ± 7 in the case of thromboembolism, 69% ± 5 in the case of AA without thromboembolism and 86% ± 6 in the case of hemolytic PNH without thromboembolism or AA	P=0.03 for thromboembolism group
Takahashi et al. (57)	AA/PNH (5 pt)	PBPC (5 pt)	HLA-matched family donor (5 pt)	Cy 60 mg/kg/d on days -7 and -6, Flu 25 mg/m^2^/d on days -5 to -1, ATG 40 mg/kg/d on days -5 to -2	DVT (3 pt)	5-month OS	100%	NA
Srinivasan et al. (58)	cPNH (12 pt), AA/PNH (14 pt)	PBPC (26 pt)	HLA identical sibling (20 pt) or parent (2 pt), single HLA-antigen mismatched sibling (4 pt)	Cy (120 mg/kg) and fludarabine (125 mg/m^2^) with or without ATG	life-threatening venous thromboses (1 pt), thromboembolic events (3 pt), thrombosis gr III-IV after HSCT (2 pt)	21-month OS	77%	NA
Pantin et al. (59)	cPNH (11 pt), AA/PNH (14 pt)	PBPC (17 pt)	MSD (17 pt)	Cy 60 mg/kg/day on days -7 and -6, Flu 25 mg/m^2^/day on days -5 to -1	3 pt with thrombotic events prior to HSCT	6-year OS	87.8%	NA
Liu et al. (60)	cPNH (15 pt), AA/PNH (29 pt)	BM + PBPC (33 pt), PBPC (8 pt), BM (3 pt)	haploidentical donor (25 pt), MSD (15 pt), MUD (4 pt)	Flu 30 mg/m^2^/d on days -7 to -2, Cy 50 mg/kg/d on days -4 to -3, and rabbit ATG 2.5 mg/kg/d on days -8 to -4 (MSD patients); Bu 3.2 mg/kg/d on days -7 and -6, Cy 50 mg/kg/d on days -5 to -2, and ATG 2.5 mg/kg/d on days -5 to -2 (MUD and haploidentical donors)	1 pt died due to transplant-associated thrombotic microangiopathy (TA-TMA)	3-year OS	90.4% ± 4.6%; 3yGFFS 85.6% ± 5.4%	NA
Sahin et al. (61)	cPNH (3 pt, AA/PNH (3 pt)	NA	NA	NA	thrombosis incidence was 1.8% per observation year in the studied group of 60 pt	3.5-year OS	83.3%	NA
Yılmaz et al. (62)	cPNH (16 pt, AA/PNH (19 pt)	BM (10 pt), PBPC (25 pt)	MUD (12 pt), MSD (23 pt)	fludarabine-based RIC: Cy, Flu, and ATG (7 pt); Bu, Flu, and ATG (28 pt)	1 patient with PNH-AA had a history of thromboembolism before transplantation	2-year OS	81.3% in cPNH and 79.9% in AA/PNH,	P=0.87
						2-year GFFS	79% in cPNH and 76% in AA/PNH	P=0.977
Nakamura et al. (63)	cPNH (42 pt)	BM (26 pt), PBPC (13 pt), cord blood (3 pt)	HLA-matched family donor (28 pt), MUD (14 pt)	Flu/Cy-based (21 pt) or Cy plus TBI or total lymphoid irradiation-based (10 pt) with ATG added (14 pt)	not specified	6-year OS	74%	NA
Markiewicz et al. (64)	cPNH (27 pt), AA/PNH (51 pt)	BM+ PBPC (1 pt), PBPC (56 pt), BM (21 pt)	MUD (49 pt), MMUD (10 pt), identical sibling (19 pt)	RTC or RIC: Treo/Flu (42 pt), Treo/Cy (9 pt)	thrombotic episodes reported in 9 pt	3-year OS	88.9% in cPNH and 85.1% in BMF/PNH	P=ns
						3-year OS	for patients with/without thrombosis, 3-year OS was 50%/92% in the cPNH group and 83.3%/85.3% in the BMF/PNH group	P=ns
						3-year OS	93.9% in BMF/PNH patients with hemolysis, 62.9% in BMF/PNH patients without hemolysis (hazard ratio, 0.13)	P=0.016
Gavrillaki et al. (65)	AA/PNH (3 pt)	BM/PBPC	MSD/MUD	Cy 50 mg/kg/day for 4 days, ATG 2.5 mg/kg/day for 3 days; patients sensitized with multiple transfusions: Cy 50 mg/kg/day for 4 days, Flu 30 mg/m^2^/day for 4 days, ATG 7.5 mg/kg and 10 mg/kg for sibling and unrelated donors, respectively, as previously described	2 pt died to thrombotic complications associated with severe acute GVHD	2-year OS	33.3%	
Halder et al. (66)	cPNH (2 pt)	unknown	MSD (2 pt)	Flu for 4 days, Cy for 2 days, and horse ATG for 4 days	none	NA	NA	NA
Andolina et al. (67)	cPNH (2 pt)	unknown	MSD twin (1 pt), MUD (1 pt)	Flu 30 mg/m^2^/day on days -5 to -2, Cy 60 mg/kg/day on days -3 to -2, and horse ATG 30 mg/kg/day on days -4 to -2; Flu 30 mg/m^2^ × 4 days on days -5 to -2, Cy 50 mg/kg × 2 days on days -3 to -2, low-dose TBI (2 Gy) × 1 on day -1, and alemtuzumab 10 mg × 4 days on days -7 to -4	none	NA	NA	NA
Salamonowicz-Bodzioch et al. (68)	AA/PNH (8 pt)	BM/PBPC	MUD/MSD	Flu 120-160 mg/m^2^5, Cy 100-200 mg/kg, and ATG Grafalon 60 mg/kg or Thymoglobulin 10 mg/kg	none	4-year OS	100% in PNH positive subgroup, 91% in PNH negative subgroup	P=ns

DFS, disease free survival; GFFS, graft versus host free; failure free survival; OS, overall survival; NA, not applicable; and ns, not significant.

Initial attempts at alloHCT have shown a high diversity in preparation regimens, or even a lack thereof. In 1979, Hershko et al. reported the disappearance of PNH after PBPCT in an identical twin donor without pretransplant conditioning (39). In contrast, syngeneic BMT without preparative marrow ablation or IST was unsuccessful in a 10-year-old girl because of the survival advantage of GPI-negative cells over transplanted stem cells and because the PNH clone progressed after BMT (41).

Until fludarabine (Flu) was introduced into clinical practice, patients with BMF syndromes underwent transplantation after receiving various busulfan (Bu) based myeloablative conditioning (MAC) regimens (69, 70). In 1985, Antin et al. reported 4 patients with severe aplastic anemia (SAA)/PNH who underwent BM transplantation from matched sibling donors (MSDs). Three patients were conditioned with cyclophosphamide, procarbazine, and antithymocyte serum (Cy/PCZ/ATS), while the other was conditioned with Bu/Cy/PCZ/ATS (42). There was no reappearance of the PNH clone up to 5 years after transplantation. In 2000, Raiola et al. presented 7 patients with PNH, aged 23–37 years, who received transplants of unmanipulated BM from MSDs (43). The conditioning regimens were Cy (160 mg/kg) and Bu (10–14 mg/kg), and all patients lived with full chimeras, complete hematologic recovery, and no evidence of PNH. In 2001, a Japanese study reported a MSD transplantation after patient consisting of cladribine (6 × 0.11 mg/kg), Bu (2 × 4 mg/kg), and rabbit ATG (2 × 2.5 mg/kg) (44). Donor chimerism of 90–100% was achieved from day 14. Lee et al. published a series of 4 patients with hypercellular marrow who received Bu-Flu-ATG (Bu 4 mg/kg/day for 2 days, Flu 30 mg/m^2^/day for 6 days, and ATG 20 mg/kg/day for 4 days) as conditioning therapy, and 1 patient with hypocellular marrow was conditioned with Cy-ATG (Cy 50 mg/kg/day for 4 days and ATG 30 mg/kg/day for 3 days) (45). Three patients received hematopoietic grafts from MSDs, and 2 patients received grafts from 1-antigen mismatched unrelated donors (mMUDs), which resulted in the engraftment of three lineages and the disappearance of a PNH clone in all 4 cases.

Since 2000, Bu-based conditioning protocols for BMF syndromes have been less commonly reported, but not completely absent. Chen et al. reported on alloHCT in 4 patients with cPNH and 14 patients with AA/PNH (46). The conditioning regimens included modified Bu (9.6 mg/kg)/Cy (3.6 g/m^2^)-based regimens for 13 patients and non-myeloablative regimens (Bu 6.4 mg/kg or Cy 100 mg/kg+Flu 150 mg/m^2^+ATG 10 mg/kg) for 5 patients. The 5-year estimated disease-free survival rate was 80.5±10.2%. A Mexican study reported on 6 patients with PNH who underwent transplantation after conditioning with Bu and Cy (47). The conditioning regimen consisted of 8 mg/kg, Cy 1050 mg/m^2^, and Flu 90 mg/m^2^ (71). An Iranian study presented 13 adult patients with PNH (5 patients with AA/PNH and 8 with cPNH) who underwent alloHCT from MSDs between 2002 and 2014. All patients received Bu-Cy as the conditioning regimen. Survival analysis revealed 5- and 13-year survival rates of 74%, and a significant relationship between a positive history of thrombosis and a high mortality rate (48). A Chinese study included 46 patients who underwent alloHCT (16 patients with PNH and 30 with AA/PNH) between 2017 and 2018 (60). The conditioning regimens were Bu (6.4 mg/kg), Cy (50 mg/kg), and ATG (10 mg/kg) in haploidentical and unrelated donors. The patients with MSDs were treated with Flu (180 mg/m^2^), Cy (100 mg/kg), or ATG (12.5 mg/kg). The estimated 3-year overall survival (OS) rates in the PNH and AA/PNH groups were 100% (±0.0) and 85.7% (±6.6), respectively (P=0.141); while the rates of GVHD-free and failure-free survival (GFFS) were 100% (±0.0) and 78.7% (±7.7), respectively (P=0.067). Bu-Cy-ATG-based conditioning was administered to 17 cases of unmanipulated haploidentical transplantations, resulting in a 3-year OS probability of 77.8% (±15.2) (50). Another Chinese study reported on 32 patients with PNH who underwent alternative donor alloHCT after Bu (8–16 mg/kg), Cy (2000–3600 mg/m^2^), or Bu (8–16 mg/kg)-Flu (120–150 mg/m^2^) (51). Two types of rabbit ATG products were used: ATG-thymoglobulin (Sanofi, Paris, France) at a total dose of 7.5–10 mg/kg in 17 patients, and ATG-fresenius (Astellas Pharma Inc., Tokyo, Japan) at a total dose of 20 mg/kg in 12 patients. Three patients were administered alemtuzumab (1 mg/kg). GVHD prophylaxis consisted of mycophenolate mofetil and methotrexate in all patients, and CsA or tacrolimus. In 31 patients, the PNH clones disappeared within 100 days of alloHCT.

Attempts to reduce conditioning toxicity have resulted in the exploration of various alternative protocols, including low-dose total-body irradiation (TBI) and treosulfan (Treo). In 2001, Hegenbert et al. reported 7 adult patients with high-risk PNH who underwent PBPCT from MSDs (n=2) and MUDs (n=5). Conditioning included Flu 30 mg/m^2^/day on days -4 to -2 and 2 Gy of TBI on day 0 (52). All 7 patients achieved durable engraftment. After 28 days, a median of 77% (range, 53–96%) T-cell donor chimerism was found in the BM and peripheral blood. T-cell chimerism increased to 91% (range, 76–100%) on day +180 and to 100% in all surviving patients after 12 months. All 7 patients achieved complete disease remission, but 3 patients died of TRM.

Cooper et al. reported 55 patients with PNH who underwent alloHCT with either MAC or reduced-intensity conditioning (RIC) regimens (53). Myeloablative regimens included a Bu dose ranging from 600 to 950 ng/mL in the serum and daily doses for 4–5 days in 18 patients; Treo 42 g/m^2^, Flu 150 mg/kg with or without 2 Gy TBI and rabbit ATG 6 mg/kg in 4 patients; and Cy 120 mg/kg with ATG 60 mg/kg and high-dose TBI (12.0–14.0 Gy) in 4 patients. The RIC regimens comprised 200 mg/kg Cy in 1 patient; 200 mg/kg Cy with 60 to 90 mg/kg ATG in 3 patients; 120 mg/m^2^ Flu, 100 mg/kg Cy, 90 mg/kg ATG, and low-dose TBI (2 Gy) in 2 patients; and 90 to 150 mg/m^2^ Flu with low-dose TBI (2.0–4.0 Gy) in 21 patients. At 5 years, the OS rate was 70%, and neither the choice of conditioning intensity (MAC vs. RIC) nor the PNH subtype (classic vs. having a clone associated with another marrow disorder) affected survival.

In a small, single-center study, Gavrillaki et al. analyzed the outcomes of 24 patients with AA who underwent transplantation between 1992 and 2018. A large PNH clone was found in 3 patients without signs of overt hemolysis or thrombosis. All of these patients achieved complete donor chimerism; however, 2 developed severe acute GVHD, which was associated with thrombotic events during the post-transplant period, leading to multiple organ failure during the early post-transplant period. The presence of PNH clones worsened the outcome of alloHCT in patients with AA (72).

Only one study has described the transplantation of T cell-depleted hematopoietic cells, it involved 5 patients with PNH (54). Conditioning consisted of thoracoabdominal irradiation (4–6 Gy), 100 mg/kg Cy, 150 mg/m^2^ Flu, and ATG or alemtuzumab, whereas graft manipulation involved alpha-beta T cell and CD19 depletion. All patients achieved full donor chimerism and survived beyond 1.7 years after HCT.

Since the 2000s, reduced intensity/toxicity and non-myeloablative protocols for AA/PNH have been explored. In 2012, the European Society for Blood and Marrow Transplantation (EBMT) registry published a retrospective study on 211 patients with PNH, reporting a 5-year OS rate of 68% (±3) in the transplanted group (54%±7 in thromboembolism cases, 69%±5 in AA without thromboembolism, and 86%±6 in hemolytic PNH without thromboembolism or AA) (56). The conditioning protocols comprised 70% Cy-based and 30% Flu-based.

Favorable outcomes of alloHCT have been reported, particularly after Flu-Cy-based conditioning regimens. In 2004, Takahashi et al. reported 5 patients with PNH who were transplanted from MSDs after conditioning with Cy and Flu; all 5 patients survived without evidence of PNH 5 to 39 months after transplantation (57). Non-myeloablative transplant conditioning consisted of Cy 60 mg/kg/day (administered on days -7 and -6), Flu 25 mg/m^2^/d (administered on days -5 to -1), and ATG 40 mg/kg/day (administered on days -5 to -2). Srinivasan et al. further studied Flu-Cy in heavily transfused patients. Twenty-six patients with transfusion-dependent/ATG-refractory SAA, PNH, or pure red cell aplasia underwent alloHCT from MSD (58). Transplant conditioning consisted of Cy (120 mg/kg) and Flu (125 mg/m^2^) with or without ATG. The post-transplantation IST consisted of CsA alone or in combination with mycophenolate mofetil or methotrexate. Twenty-four of the 26 patients survived for a median 21 months after transplantation.

Pantin et al. investigated 17 patients with cPNH or AA/PNH (59). Patients received PBPCT from 6/6 MSDs after conditioning with Cy 60 mg/kg/day (days -7 and -6) and Flu 25 mg/m^2^/day (days -5 to -1). To prevent graft rejection, patients with a significant transfusion history had horse ATG (40 mg/kg/day, days -5 to -2) added to the conditioning regimen (n = 14). Over a median follow-up of nearly 6 years, 15 patients (87.8%) survived without any evidence of PNH, transfusion dependence, or anticoagulation agent use.

Chinese study analyzed the outcomes of 44 patients with PNH who underwent alloHCT from haploidentical donors (25), MSDs (15), and MUDs (4) (49). Patients with MSDs were administered a Flu/Cy-based regimen comprising Flu 30 mg/m^2^/day intravenously (i.v.) on days -7 to -2, Cy 50 mg/kg/day i.v. on days -4 to -3, and rabbit ATG (Thymoglobulin; Genzyme) 2.5 mg/kg/day i.v. on days -8 to -4. Patients with MUDs or haploidentical donors received a Bu/Cy-based regimen consisting of Bu 3.2 mg/kg/day i.v. on days -7 and -6, Cy 50 mg/kg/day i.v. on days -5 to -2, and ATG 2.5 mg/kg/day i.v. on days -5 to -2. The probabilities of 3-year OS and GFFS were 90.4%±4.6% and 85.6%±5.4%, respectively. The 3-year OS rates of the haplo-donor and MSD groups were 86.5%±7.3% and 93.3%±6, respectively (P=0.520). The 3-year GFFS rates of the haploidentical and MSD groups were 78.3%±8.6 and 92.9%±6.9, respectively (P=0.250).

In a Turkish study, the transplant outcomes of 35 patients with PNH (16 with cPNH and 19 with AA/PNH) after low-intensity conditioning were investigated (62). The most common conditioning protocol for the 24 patients was Flu-Cy. Rabbit ATG was administered in 29 patients, and horse ATG was used in six patients. AlloHCT was performed using MUDs and MSDs in 12 and 23 patients, respectively. The 2-year OS and GFFS rates were 81.2% and 78.1%, respectively. The 2-year OS rates in patients with cPNH and AA/PNH were 81.3% and 79.9%, respectively (P=0.87), and the 2-year GFFS rates in patients with cPNH and AA/PNH were 79% and 76%, respectively (P=0.977).

A Japanese study involving 42 adult patients with PNH who underwent alloHCT reported a 6-year OS rate of 74% (63). The conditioning regimens varied widely; the most commonly used regimens were Flu/Cy-based (N=21) and Cy plus TBI or total lymphoid irradiation (N=10), with ATG administered to 14 patients. The leading cause of death was graft failure, which occurred in 11 patients. Younger age (<30 years) and fewer packed red cell blood transfusions (<20) were associated with improved survival.

One of the largest studies on alloHCT in AA-PNH/PNH was published by the Polish study group (64). This retrospective analysis of patients with PNH who underwent transplantation between 2002 and 2016 included 78 patients, with 27 with cPNH and 51 with AA/PNH. The median age in the group was 29 years old (range, 12 to 65 years). There was a history of thrombosis in 12% and a history of hemolysis in 81%, and 92% of the patients required erythrocyte transfusions before undergoing alloHCT. In 66% of the cases, treosulfan-based conditioning was used, and in 72% of the cases, HCT was performed from a MUD. The 3-year OS rates of patients with cPNH and BMF/PNH were 88.9% and 85.1% (p=ns), respectively. For patients with/without thrombosis, the 3-year OS was 50%/92% (p=ns) in the cPNH group and 83.3%/85.3% (p=ns) in the BMF/PNH group. In the BMF/PNH patients with/without hemolysis, the 3-year OS was 93.9%/62.9% (hazard ratio, 0.13; P = 0.016).

Few studies have included pediatric patients with PNH. An Indian study reported 52 pediatric patients with PNH, 20 with cPNH and 32 with AA/PNH (66). Ten of the 20 (50%) patients received danazol for the treatment of cPNH, and 6 were treated with ATG for AA/PNH. Only two patients with cPNH underwent matched sibling alloHCT after Flu-Cy-horse ATG conditioning. Andolina et al. reported 2 children with PNH who received transplantation after Flu 30 mg/m_2_/day on day -5, Cy 60 mg/kg/day on day -3, and horse ATG 30 mg/kg/day on day -4, or Flu 30 mg/m^2^ × 4 days on day -5, Cy 50 mg/kg × 2 days on day -3, low-dose TBI (2 Gy) × 1 on day -1, and alemtuzumab 10 mg × 4 days starting on day -7 (67). Salamonowicz-Bodzioch et al., studied 56 children with SAA, among these, PNH clones were identified in 8, and all patients underwent alloHCT from MUDs or MSDs. The patients received transplantation after a conditioning protocol comprising Flu (120–160 mg/m^2^), Cy (100–200 mg/kg), and ATG Grafalon (60 mg/kg) or Thymoglobulin (10 mg/kg). The estimated OS was similar between the PNH-positive and PNH-negative subgroups (100% vs. 91%, not significant).

## Discussion

Despite awareness of the prevalence of PNH clones in patients with BMF syndromes, an optimal approach for patients with this type of presentation has not been established. Patients with nonclassic PNH are treated according to the dominant hematological manifestations, typically AA or MDS with IST and/or alloHCT. The presence of a PNH clone may be associated with favorable prognosis in cases of IST and alloHCT, but it is not considered relevant for routine therapeutic strategies (23, 73). However, this model is incomplete and has limitations that need to be addressed in the future, such as cytopenic manifestations that do not fit into the SAA or MDS categories, and a lack of a clear connection between the size of the PNH clone and the severity of hematological symptoms (13). The evolution of the SAA and AA/PNH conditioning regimens over the last 40 years has resulted in changes from myeloablative to non-myeloablative and more AA-targeted protocols, with improvements in survival rates and decreased toxicities. Reducing the intensity of preparative regimens could allow alloHCT to be offered to patients with PNH with less favorable characteristics (74). The best available conditioning protocol for AA/PNH is yet to be determined; however, according to real-world data, Flu-Cy protocols with ATG serotherapy should be considered in most cases (23). The use of Treo in patients with SAA and PNH clones may improve overall transplant outcomes by reducing toxicity and supporting engraftment; however, Treo is not specifically targeted towards PNH clone management. The clinical decision to use Treo in SAA with a PNH clone should focus more on the patient's overall risk profile and tolerance to conditioning agents rather than the presence of PNH clones (75). Currently, there are no well-defined guidelines specifying the minimum PNH clone size that would indicate a preference for Treo over high-dose Cy combinations with Flu and ATG in conditioning regimens. However, recent and ongoing studies (NCT04965597) have suggested that a larger PNH clone, typically ≥10%, may prompt physicians to consider Treo because of its reduced risk of conditioning-related toxicities. This is especially advantageous given the increased thrombotic risk in adult patients with PNH. Regarding the consensus on SAA therapy in children, independent of PNH status, the conditioning protocol should comprise Flu (30 mg/m^2^/day, ×4 days) and Cy (25 mg/kg/day, ×4 days) with ATG serotherapy (76). Higher BM cellularity, along with a larger clone size, should direct towards use of more myeloablative protocols. In the case of cPNH, an acceptable conditioning protocol can be based on a Treo-Flu backbone (64).

Another issue to be addressed is whether patients with cPNH would benefit from alloHCT, and whether this therapeutic option should be offered to them in clinical trials. Retrospective studies are not sufficient to address this research question conclusively because they date from the pre-eculizumab era or are based in countries with limited access to C5 inhibitors. Furthermore, in most cases, patients with cPNH are not clearly identified or constitute only a marginal subgroup, thereby evading proper analysis. Significant early transplant-related toxicities in cPNH and unresolved issues in long-term complications such as post-transplant quality of life and incidence of chronic GVHD, have not been adequately addressed in retrospective studies. Moreover, current improvements in transplantation techniques and supportive care may affect alloHCT outcomes in patients with cPNH. One can hypothesize that the financial burden of anti-complement therapy in cPNH may limit the initiation of clinical studies, especially in middle-income countries that have developed transplantation programs. There are no clear data on the duration of complement inhibitor administration. However, the incorporation of a short course of anticomplement treatment in the peri-transplant and early post-transplant periods should be considered in the prospective transplantation protocols for patients with overt IVH or a clinically significant PNH clone to decrease the risk of IVH-related thromboembolism and complications resulting from endothelial injury (72, 77). In some centers, administration of eculizumab is stopped during the conditioning regimen, whereas in others it continues up to engraftment (53, 78). Indications for alloHCT in patients with cPNH might include transfusion-dependent cPNH with no access to complement-targeted therapy or anti-complement therapy inefficacy (53, 79).

Furthermore, the dilemma of whether to offer alloHCT to patients with PNH and recurrent thromboembolic events should be addressed because, according to the reported data, this translates into worse alloHCT outcomes (64). In the era of novel and highly efficient therapies targeting the proximal complement pathway, the identification of high-risk patients is controversial. If patients with PNH and a thromboembolic history must be subjected to alloHCT, the need for continuing anti-complement therapy has been suggested, which reduces the risk of post-transplant thrombotic events and improves post-transplantation survival rates (53, 79–81). However, data concerning complement inhibitor use are limited, and they may be recommended until red cell engraftment and PNH clone elimination.

## Conclusions

Although it was not an objective of this study to provide recommendations for alloHCT in PNH, future transplantation studies should generally consider choosing less toxic transplant conditioning regimens according to the dominant manifestation, and must differentiate cytopenia with aplastic BM and an asymptomatic PNH clone from cPNH with a dominant risk of IVH-related complications. Immunoablative protocols based on ATG serotherapy with Flu and Cy are recommended for the alloHCT of SAA/AAs patients with associated PNH clones (23). In patients with cPNH, the tendency to use reduced toxicity protocols with Flu has been well documented; however, there is no consensus on the optimal efficacy of drugs in the conditioning protocol. A Treo/Flu-based reduced-toxicity conditioning regimen can be recommended for cPNH as an alternative to Bu-based protocols. In cPNH with a significant disease burden, the decision to proceed with alloHCT in the era of proximal complement pathway inhibitors should be carefully evaluated, with consideration of the costs and resources, treatment availability, and the clinical presentation of each patient. Concomitant peri-transplant administration of C5 inhibitors can be considered in such cases (53, 79, 80). However, in the absence of randomized clinical trials comparing complement inhibitors and alloHCT, retrospective studies provide limited guidance.
